# Noggin Inhibits IL-1β and BMP-2 Expression, and Attenuates Cartilage Degeneration and Subchondral Bone Destruction in Experimental Osteoarthritis

**DOI:** 10.3390/cells9040927

**Published:** 2020-04-10

**Authors:** Szu-Yu Chien, Chun-Hao Tsai, Shan-Chi Liu, Chien-Chung Huang, Tzu-Hung Lin, Yu-Zhen Yang, Chih-Hsin Tang

**Affiliations:** 1 Department of Exercise Health Science, National Taiwan University of Sport, Taichung 404393, Taiwan; u100054860@cmu.edu.tw; 2 School of Medicine, China Medical University, Taichung 404022, Taiwan; u104054003@cmu.edu.tw; 3 Department of Orthopedic Surgery, China Medical University Hospital, Taichung 404022, Taiwan; ritsai8615@gmail.com; 4 Department of Sports Medicine, College of Health Care, China Medical University, Taichung 404022, Taiwan; 5 Department of Medical Education and Research, China Medical University Beigang Hospital, Yunlin 651012, Taiwan; sdsaw.tw@yahoo.com.tw; 6 Division of Immunology and Rheumatology, Department of Internal Medicine, China Medical University Hospital, Taichung 404022, Taiwan; 7 Material and Chemical Research Laboratories, Industrial Technology Research Institute, Hsinchu 310401, Taiwan; DustyLin@itri.org.tw (T.-H.L.); Itri534604@itri.org.tw (Y.-Z.Y.); 8 Graduate Institute of Biomedical Science, China Medical University, Taichung 404022, Taiwan; 9 Chinese Medicine Research Center, China Medical University, Taichung 404022, Taiwan; 10 Department of Biotechnology, College of Health Science, Asia University, Taichung 404, Taiwan

**Keywords:** osteoarthritis, interleukin-1β, bone morphogenetic protein 2, cartilage remodeling

## Abstract

Osteoarthritis (OA) is a chronic inflammatory and progressive joint disease that results in cartilage degradation and subchondral bone remodeling. The proinflammatory cytokine interleukin 1 beta (IL-1β) is abundantly expressed in OA and plays a crucial role in cartilage remodeling, although its role in the activity of chondrocytes in cartilage and subchondral remodeling remains unclear. In this study, stimulating chondrogenic ATDC5 cells with IL-1β increased the levels of bone morphogenetic protein 2 (BMP-2), promoted articular cartilage degradation, and enhanced structural remodeling. Immunohistochemistry staining and microcomputed tomography imaging of the subchondral trabecular bone region in the experimental OA rat model revealed that the OA disease promotes levels of IL-1β, BMP-2, and matrix metalloproteinase 13 (MMP-13) expression in the articular cartilage and enhances subchondral bone remodeling. The intra-articular injection of Noggin protein (a BMP-2 inhibitor) attenuated subchondral bone remodeling and disease progression in OA rats. We also found that IL-1β increased BMP-2 expression by activating the mitogen-activated protein kinase (MEK), extracellular signal-regulated kinase (ERK), and specificity protein 1 (Sp1) signaling pathways. We conclude that IL-1β promotes BMP-2 expression in chondrocytes via the MEK/ERK/Sp1 signaling pathways. The administration of Noggin protein reduces the expression of IL-1β and BMP-2, which prevents cartilage degeneration and OA development.

## 1. Introduction

Osteoarthritis (OA) is a chronic inflammatory condition characterized by the progressive, degenerative disease of the joints and is highly prevalent in the elderly, in obese patients, and in patients with joint trauma [1,2,3,4]. Severe OA is associated with a poor health-related quality of life and substantial interference with productivity [5]. Patients with OA experience progressive degeneration of the articular cartilage, chondro-osteophyte formation (new formation of ectopic cartilage and bone in the joint), subchondral bone remodeling (sclerosis), synovitis, and infiltration of inflammatory cells in the joint capsule [1,2,6,7]. Although tissues throughout the OA joint are involved in the inflammatory process during disease progression, irreversible destruction of the articular surface and increased subchondral bone turnover are the primary features of OA joints [8,9]. In particular, abnormal tissue production and changes in the subchondral bone are often observed around the degraded cartilage. It is thought that subchondral bone remodeling occurs in response to joint damage, resulting in osteophytes at the joint margins [6]. Increasingly, research is exploring alterations in the subchondral bone area as possible mediators of the structural disease progression in OA. Subchondral bone remodeling is a key factor in the development of OA in several experimental OA models [8,10]. Osteochondral changes occur in the early-stage of OA and may aggravate the OA pathology of other tissues in the joint [3,4]. The loss of osteochondral integrity removes the barrier between intra-articular and subchondral compartments, exposing subchondral bone and other tissues to abnormal chemical and biochemical influences [11]. Importantly, despite what is known about OA, its pathology remains unclear and no treatments are able to stop or reverse the progression of this disease [12,13].

The inflammatory cytokine interleukin 1 beta (IL-1β) and its receptors IL-1 receptor type I (IL-1RI), IL-1 receptor type II (IL-1RII), and IL-1 receptor antagonist (IL-1RA) belong to the IL-1 family [14]. The IL-1 family members are primarily associated with innate immunity and inflammation. They signal through binding to IL-1RI but are neutralized via binding to IL-1RII or IL-1RA [14,15]. IL-1β plays a crucial role in both local inflammatory responses and systemic acute responses [14,16]. Moreover, IL-1β stimulates the release of other proinflammatory cytokines, such as IL-6 and tumor necrosis factor alpha (TNF-α) [16]. Elevated levels of IL-1β are associated with tissue damage and reflect the severity of inflammation. A reduction in IL-1β levels is associated with a reduction in the inflammatory process; this relationship has been found in various different organs, such as the brain [15], pancreas [16], colon [17], and heart [18]. Both low-level and chronic inflammation contribute to OA development and progression [11,13,19]. High levels of IL-1β expression are found in the synovial membrane, cartilage, subchondral bone, and also the synovial fluid of OA patients [20,21,22]. IL-1β is capable of inducing inflammatory reactions independently and in combination with other degradation mediators in OA [11,23]. IL-1β influences the production of cartilage-degrading enzymes (matrix metalloproteinases (MMPs)), inhibits extracellular matrix (ECM) synthesis, and stimulates the expression of other proinflammatory cytokines in the OA tissue, both in vitro and in vivo [9,20]. The stimulation of IL-1β induces progressive proliferation and hypertrophy of chondrocytes in the OA cartilage [24]. IL-1β also induces the promineralizing activity in chondrocytes and catabolic effects in the bone, which may in turn promote cartilage calcification (ossification) and degeneration involved in the progression of subchondral bone remodeling [21,25,26].

Macrophages play a crucial role in the maintenance and recovery of tissue homeostasis [27,28]. Macrophage functions depend on some level of inflammation, which is necessary for the repair of tissue after tissue damage incurred by infection or inflammation [27,28,29]. Two major macrophage subpopulations with different functions include the classically activated or inflammatory (M1) and alternatively activated or anti-inflammatory (M2) macrophages. Both play major roles in the resolution of inflammation [27,28]. The balanced polarization between M1 and M2 macrophages seems to be impaired in chronic inflammation, such as in OA [28,29], which is characterized by inflammation and simultaneous attempts by the body to repair ongoing injury [30]. Ongoing research is exploring the mechanisms of macrophage polarization and their role in health and disease [28,30]. Various explanations have been proposed concerning how inflammation causes the calcification of the cartilage. Remarkably, some characteristics of OA are similar to the processes that occur in endochondral ossification during skeletal development, such as the hypertrophic differentiation of chondrocytes, remodeling and mineralization of the cartilage, and apoptotic deaths of chondrocytes [10,25,26]. Recent research has indicated a role for bone morphogenetic protein (BMP) signaling in destructive, remodeling, and balanced arthritis [31]. It is known that endochondral ossification is influenced by BMPs, members of the transforming growth factor beta (TGF-β) superfamily [32]. Chondrocytes have the ability to produce BMPs and to induce and promote new cartilage and bone formation at an ectopic site, so they play an important role in bone growth and development. Targeting BMP-2 is considered to be a strategy for cartilage repair and chondrogenesis [33,34]. Whereas, levels of BMP-2 are negligible in normal, healthy cartilage, they are high in the OA cartilage [35,36]. BMP-2 has potent anabolic actions, but can also lead to the degradation of aggrecan and trigger OA-like cartilage changes by interacting with other degrading enzymes [32,36]. Notably, multiple intra-articular injections of BMP-2 induce significant osteophyte formation in the murine knee joint [37]. This suggests that BMP-2 is associated with reparative responses, as well as pathological side effects that cause cartilage damage. Evidence from murine OA models and OA human cartilage samples has revealed that levels of BMP-2 expression increase in the cartilage and osteophytes according to the severity of the OA disease [35,36,38]. Stimulation of proinflammatory cytokines increases the production of active BMP-2 in both cellular and tissue explant culture from the OA cartilage [39]. The secretion of these factors from chondrocytes has been implicated in the alteration of the biochemical and functional abilities of the subchondral bone, and stimulation of the subchondral area remodeling process [40]. Based on this evidence, we hypothesized that IL-1β is involved in BMP-2 expression and plays important roles in cartilage degradation and structural remodeling.

Noggin was initially identified as a dorsalizing agent in *Xenopus laevis* and is expressed in a number of tissues [41]. As an extracellular antagonist of BMP, Noggin binds directly to various BMPs based on different affinities, such as BMP-2, -4, -7, -13, and -14 [41,42,43]. This interaction prevents BMPs from binding to their cell surface receptors, disabling the initiation of BMP signaling in target cells and regulating BMP activity in many body tissues [43,44]. The BMP/Noggin interaction is important for normal embryonic development [44]. Noggin is involved in developmental structures derived from ectoderm and plays a critical role in the development of the neural tube, teeth, hair follicles, and the eye [43], as well as embryonic chondrogenesis, osteogenesis, and joint formation [45]. Mice lacking Noggin exhibit excessive BMP activity, severe defects in somitogenesis, and skeletal malformation [43,45]. In a cohort of patients with ankylosing spondylitis, an imbalance between BMP-2 and Noggin expression was characterized by bone resorption and formation during bone remodeling, exhibited as pathologic osteogenesis [46].

Our findings in this study suggest that exogenous IL-1β stimulates BMP-2 expression in chondrocytes. Our investigation into the signaling pathway underlying BMP-2 upregulation revealed that IL-1β promotes BMP-2 expression in a culture medium via the mitogen-activated protein kinase (MEK), extracellular signal-regulated kinase (ERK), and specificity protein 1 (Sp1) signaling pathways. The intra-articular injection of BMP-2 inhibitors attenuated articular cartilage degradation and subchondral bone destruction in the experimental OA rat model. Our findings indicate that proinflammatory cytokines increase BMP-2 expression in chondrocytes and participate in pathological changes of the subchondral bone in OA. Our findings may explain the mechanism of joint structure destruction during chronic inflammation in OA.

## 2. Materials and Methods

### 2.1. Materials

The IL-1β antibody (bs-6319R-TR) was purchased from Bioss Inc. (Boston, MA, USA) and the BMP-2 antibody (18933-1-AP) was purchased from Proteintech (Wuhan, Hubei, China). The phospho-MEK1/2 (Ser221) (166F8) (pMEK1/2) antibody (2338) was purchased from Cell Signaling Technology (Danvers, MA, USA). The phospho-Sp1 (phospho-Thr453) (pSp1) antibody (ab59257) was purchased from Abcam (Cambridge, MA, USA). Anti-Sp1 (GTX110593) was purchased from GeneTex (Hsinchu City, Taiwan). The β-actin antibody (A5441) was purchased from Sigma-Aldrich (St. Louis, MO, USA). Antibodies against phosphorylated ERK (Tyr204) (pERK) (sc-7383), ERK2 (sc-1647), MEK1 (sc-6250), and MMP-13 (sc-515284) were purchased from Santa Cruz (Santa Cruz, CA, USA). The recombinant mouse IL-1β/IL-1F2 (401-ML) and mouse Noggin protein (1967-NG) were purchased from R&D Systems (Minneapolis, MN, USA). MEK inhibitors PD98059 (P215) and U0126 (U120), and the selective Sp1 inhibitor, mithramycin A (530310), were purchased from Sigma-Aldrich (St. Louis, MO, USA). The ERK-selective inhibitor, FR180204 (sc-203945), was purchased from Santa Cruz (Santa Cruz, CA, USA). The DharmaFECT 1 transfection reagent (T-2001), ON-TARGETplus SMARTpool duplex small interfering RNAs (siRNAs) targeting Sp1, and ON-TARGETplus nontargeting control pool, were purchased from Dharmacon (Lafayette, CO, USA). The MEK1 (s77053) and ERK2 (s77104) Silencer Select predesigned siRNAs were purchased from Ambion (Austin, TX, USA). Dulbecco’s modified Eagle’s medium (DMEM) and Ham’s F12 (F12) cell culture medium were purchased from Gibco (Thermo Fisher Scientific, Waltham, MA, USA), and culture supplements were purchased from Invitrogen (Thermo Fisher Scientific). All other chemicals not mentioned above were supplied by Sigma-Aldrich (St. Louis, MO, USA).

### 2.2. ATDC5 Cell Line and Culture Conditions

The mouse chondrocytic cell line ATDC5 was purchased from the American Type Culture Collection (ATCC, Manassas, VA, USA). According to the procedure described in our previous reports [47,48], cells were maintained at 37 °C with 5% CO_2_, in a 1:1 mixture of DMEM and F12 medium containing 5% (v/v) FBS and 1% penicillin–streptomycin (all from Gibco, Thermo Fisher Scientific), until the culture reached 80% confluence.

### 2.3. Analysis of the Gene Expression Omnibus (GEO) Database

The BMP-2 gene expression profile data obtained from male rat (*Rattus norvegicus*) articular chondrocytes were downloaded from the GEO database (Profile GDS2472; Reference Series GSE6119). The Student’s *t*-test compared the BMP-2 gene expression between untreated chondrocytes and those treated with IL-1β.

### 2.4. Western Blot Analysis

The Western blot analysis was performed as described previously [47,48]. In brief, proteins extracted from the cell lysis buffer were separated by SDS-PAGE and transferred to Immobilon^®^-P PVDF membranes (Merck Millipore, Burlington, MA, USA). After blocking the membranes in 5% nonfat milk, they were then incubated with a primary antibody (rabbit polyclonal antibody against BMP-2, MMP-13, Sp1 or p-Sp1; rabbit monoclonal antibody against pMEK1/2; mouse monoclonal antibody specific for pERK, ERK2, MEK1, or β-actin) overnight at 4 °C. Then, the blots were visualized by enhanced chemiluminescence using an ImageQuant LAS 4000 camera (GE Healthcare, Little Chalfont, UK).

### 2.5. mRNA Quantification by Real-Time Quantitative Polymerase Chain Reaction (qPCR) Amplification

Total RNA was extracted from ATDC5 cells using the TRIzol reagent (MDBio, Taipei, Taiwan), according to the manufacturer’s instructions. The RNA concentration was determined using a Nanovue^TM^ spectrophotometer (GE Healthcare). RNA was converted into a complementary DNA (cDNA) by M-MLV reverse transcription (Invitrogen, Thermo Fisher Scientific), following the manufacturer’s instructions. The qPCR analysis was performed as per the manufacturer’s protocols [49,50,51,52]. cDNA was amplified with the forward (F) and reverse (R) primers by PCR. The primer sequences for mouse BMP-2 were 5′-CACACAGGGACACACCAACC-3′ (F) and 5′-CAAAGACCTGCTAATCCTCAC-3′ (R) [53]. The primer sequences for mouse GAPDH were 5′-TGTGTCCGTCGTGGATCTGA-3′ (F) and 5′-TTGCTGTTGAAGTCGCAGGAG-3′ (R) [54].

### 2.6. Enzyme-Linked Immunosorbent Assay (ELISA)

To measure the concentration of BMP-2 in the conditioned medium, the ELISA assay was performed using the methods described in our previous work [47]. The production of BMP-2 by ATDC5 cells in the conditioned medium was assayed using a mouse BMP-2 ELISA kit (Aviva Systems Biology, OKEH00026), following the manufacturer’s procedures.

### 2.7. Cell Transfection

ATDC5 cells were transfected with siRNAs targeting MEK1, ERK2, Sp1, or the nontargeting control using the DharmaFECT 1 transfection reagent (Dharmacon), according to the manufacturer’s recommendations. Briefly, the appropriate transfection medium was prepared in a serum-free medium and placed in a 6-well plate. After 24 h of transfection, the culture medium was replaced and the cells were incubated under different conditions (with or without IL-1β) for 24 h. After 48 h of transfection, BMP-2 expression was examined by qPCR and ELISA assays.

### 2.8. Inhibitor Treatment

ATDC5 cells were maintained in 6-well plates until growth reached 80% confluence, after which time the culture medium was replaced with a medium containing 1% FBS. After 24 h of incubation, the culture medium was replaced again and cells were treated with different inhibitors for 30 min (U0126, PD98059, or FR180204) or 2 h (mithramycin A) before being stimulated with IL-1β (10 ng/mL), according to the methods described in previous studies [48,55,56]. The final working concentrations were as follows: MEK inhibitors, U0126 (15 μM) and PD98059 (10 μM); ERK inhibitor, FR180204 (3 μM); Sp1 inhibitor, mithramycin A (0.3 μM).

### 2.9. Chromatin Immunoprecipitation (ChIP) Assay

Nucleoprotein complexes were prepared from ATDC5 cells treated with different doses of IL-1β (0–30 ng/mL). ChIP assays were performed using the SimpleChIP^®^ Enzymatic Chromatin IP kit (Cell Signaling Technology, Danvers, MA, USA), according to the manufacturer’s protocols. In brief, after the ATDC5 cell preprocessing (fixed and DNA-protein crosslinks) at room temperature (RT), the DNA-protein complex in cells was randomly digested with a micrococcal nuclease. Chromatin fragments were immunoprecipitated (IP) with anti-Sp1 (GeneTex, Hsinchu City, Taiwan) or normal anti-rabbit IgG as a negative control (Cell Signaling Technology, Danvers, MA, USA). Purified DNA from IP was analyzed by PCR with primers specific to the BMP-2 promoters (forward: 5′-TCACACTCATCCGGGACGC-3′; reverse: 5′-GAACACCTCCCCCTCGGA-3′) [57]. Each PCR product was analyzed by 2% agarose gel electrophoresis and visualized under ultraviolet (UV) light.

### 2.10. Experimental OA of Anterior Cruciate Ligament Transaction (ACLT) in Rat Model

Twenty-nine four-week-old male Sprague-Dawley rats were purchased from the National Laboratory Animal Center (Taipei, Taiwan) and maintained under conditions complying with the Guidelines of the Institutional Animal Care and Use Committee of China Medical University, Taichung, Taiwan. All animal procedures were approved before being performed (approval number: CMUIACUC-2018-276). The rats were randomly divided into four groups: Control (n = 5), ACLT (n = 8), ACLT+30 ng Noggin protein (ACLT+30) (n = 8), or ACLT+100 ng Noggin protein (ACLT+100) (n = 8). Experimental OA was induced by surgical transection of the right anterior cruciate ligament in the ACLT groups, using a procedure modified from previously published research [58,59]. Following surgery, the joint surface was washed with sterile saline, then the capsule and skin were sutured. After surgery (day 0), ampicillin (50 mg/kg body weight) was administered by a subcutaneous injection once a day for five days according to the previous research [59,60]. Ampicillin is a penicillin antibiotic that is used to treat or prevent many different types of infections; its use in rats is associated with very low levels of acute toxicity and demonstrates good safety [61,62]. At day 6, 50 μL of the sterile phosphate-buffered saline (PBS) containing 30 ng (0.6 μg/mL) or 100 ng (2 μg/mL) Noggin protein (BMP-2 inhibitor) [44,63,64] was administered by intra-articular injection into the ACLT-operated knee in the ACLT+30 group or the ACLT+100 group. The injections were performed twice a week for seven weeks [58]. The same volume of sterile BPS was simultaneously injected into the controls. Doses and frequencies of injections were modified based on previous reports [42,65].

### 2.11. Micro-Computed Tomography (Micro-CT) Imaging

Isolated rat tibias and femurs were fixed in 4% paraformaldehyde and then 70% ethanol. Samples were scanned using the Bruker SkyScan 2211 nano-CT (Bruker micro-CT, Knotich, Belgium) at the resolution of 8.5 μm. Micro-CT was performed using cameras that scanned over 180 degrees of rotation, with a voltage of 90 kVp, a current of 450 μA (8-Watt output), and a 0.5 mm aluminum (Al) filter to prevent beam hardening artifacts. Image reconstruction was performed using the Instarecon reconstruction software (Bruker-micro-CT, Kontich, Belgium), which also performed the ring artifact and beam-hardening correction.

### 2.12. Micro-CT Analysis

The analysis strategy was modified according to previous studies [66,67,68]. In brief, reconstructed cross-sections were re-orientated and we selected regions of interest showing an irregular anatomical contour in the subchondral trabecular regions of the medial tibial plateau. We analyzed the volume of interest areas using 0.5 mm images (59 slices). Thresholding, the selection of regions of interest, bone morphometric analysis (trabecular bone volume fraction (BV/TV); bone surface density (BS/TV); trabecular thickness (Tb.Th); trabecular number (Tb.N); trabecular separation (Tb.Sp)), and volumetric bone mineral density (vBMD) analysis was performed using the CTAn software (Version 1.7.1, Bruker micro-CT, Kontich, Belgium). BV/TV was considered to be a primary variable. Three-dimensional (3D) visualization reconstructions were performed in the proximal tibias using the CTVox software (Version 3.3.0, Bruker micro-CT, Kontich, Belgium).

### 2.13. IHC (Immunohistochemistry) Staining

IHC staining was performed as described in our previous studies [47,48]. In brief, the sections were incubated with primary antibodies specific for mouse IL-1β, BMP-2, and MMP-13. The immunodetection assay was performed using a NovoLink Polymer Detection Systems kit (Leica Biosystems, Wetzlar, Germany), following the manufacturer’s procedures. We also used Safranin O/Fast Green staining to evaluate the cartilage structure lesion of specimens from ACLT rat knees. Slides were observed under a light microscope and digitized with a Pannoramic scanner digital microscope (3DHISTECH, Budapest, Hungary). Images were obtained from digitized slides with the CaseViewer software (v 2.3, 3DHISTECH, Budapest, Hungary).

### 2.14. Statistical Analysis

All statistical analyses were performed using SPSS (v20, IBM, Armonk, NY, USA). The results are presented as the mean ± standard deviation (SD) of at least three independent experiments. The statistical comparisons of two groups were performed with the Student’s *t*-test or the Mann–Whitney *U* test, based on the analysis of the normal distribution. Statistical comparisons of more than two groups were performed using one-way analysis of variance (ANOVA) with Bonferroni or Dunnett *post hoc* testing. For all tests, *p*-values of less than 0.05 were considered to be statistically significant.

## 3. Results

### 3.1. Elevated BMP-2 Expression in Experimental OA Cartilage

**Figure 1:**
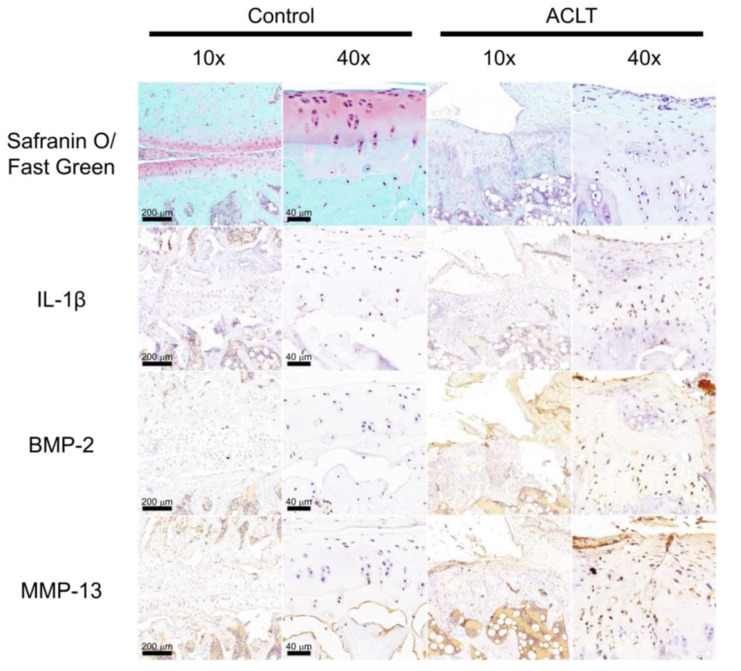
High levels of IL-1β, BMP-2, and MMP-13 expression were seen in the experimental OA model. Paraffin sections of the knee articular cartilage from the Control and ACLT groups were stained with Safranin O/Fast Green, and IHC-stained with anti-IL-1β, anti-BMP-2, and anti-MMP-13 antibodies (n = 4). Scale bar, 200 μm (10×); 40 μm (40×). Original magnification 63×. Images were obtained from digitized slides with the CaseViewer software. Abbreviations: IL-1β: Interleukin 1 beta; BMP-2: Bone morphogenetic protein 2; MMP-13: Matrix metalloproteinase 13; OA: Osteoarthritis; ACLT: Anterior cruciate ligament transaction; IHC: Immunohistochemistry.

Previous research has reported high levels of BMP-2 expression in the knee OA intra-articular tissue [35,38,69]. To confirm that this phenomenon occurs in our OA animal model, we first studied the difference in histology between control rats and ACLT rats. Compared with control rats, we found that ACLT rats had a significant articular cartilage degradation and destruction of the knee articular surface (according to Safranin O/Fast Green staining) and significantly increased expression of IL-1β, BMP-2, and MMP-13 (according to IHC staining) (Figure 1).

### 3.2. IL-1β Stimulates BMP-2 Expression in Chondrocytes

**Figure 2:**
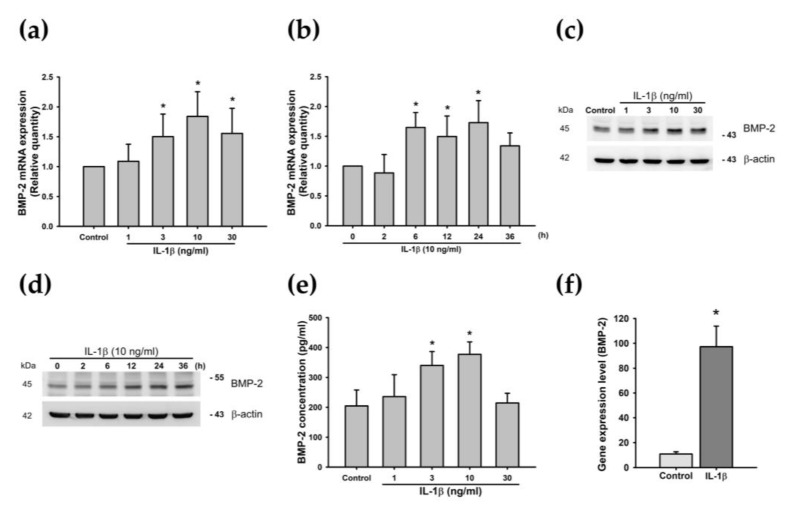
IL-1β induces BMP-2 expression in chondrocytes. (**a**,**b**) ATDC5 cells were incubated with different doses of IL-1β (0, 1, 3, 10, or 30 ng/mL) for 24 h, or with IL-1β (10 ng/mL) for the indicated times (0, 2, 6, 12, 24, or 36 h), and BMP-2 mRNA expression was evaluated by qPCR (both n = 6). Statistical testing was performed by one-way ANOVA. (**c**,**d**) ATDC5 cells were incubated under different concentrations of IL-1β for 24 h, or for the indicated times, and BMP-2 protein expression was evaluated by Western blotting (both n = 6). (**e**) ATDC5 cells were incubated with different doses of IL-1β (0, 1, 3, 10, or 30 ng/mL) for 24 h, and BMP-2 protein secretion was evaluated by ELISA (n = 6). Statistical testing was performed by one-way ANOVA. (**f**) IL-1β induced BMP-2 expression in the articular cartilage; BMP-2 gene expression was obtained from the GEO database. Statistical testing was determined by the Student’s *t*-test. Quantification results are expressed as the mean ± SD. * *p* < 0.05 compared with the Control group in (**a**,**e**,**f**), and compared with the 0 h group in (**b**). Abbreviations: IL-1β: Interleukin 1 beta; BMP-2: Bone morphogenetic protein 2; qPCR: Real-time quantitative polymerase chain reaction; GEO database: Gene Expression Omnibus database.

Inflammatory cytokines such as IL-1β and TNF-α reportedly stimulate BMP-2 expression in the OA articular cartilage [39,70]. To determine whether BMP-2 is produced in chondrocytes after the IL-1β treatment, we incubated ATDC5 cells with IL-1β at different concentrations for varying amounts of time. We observed that the treatment of chondrocytes with IL-1β increased BMP-2 mRNA expression and protein secretion in concentration- and time-dependent manners (Figure 2a–e). Although BMP-2 expression was increased from baseline at 36 h in IL-1β-stimulated cells, the level was not significantly different from that of the control group at baseline (*p* = 0.062). We selected 24 h for the time of IL-1β incubation in the following studies. Similar results are shown in our analysis of mRNA expression profiles from the GEO database, with higher levels of BMP-2 expression in IL-1β-treated chondrocytes (Figure 2f). These observations suggest that an association exists between IL-1β and BMP-2 in OA.

### 3.3. The MEK/ERK Signaling Pathway is Involved in IL-1β-Induced Stimulation of BMP-2 Expression

**Figure 3:**
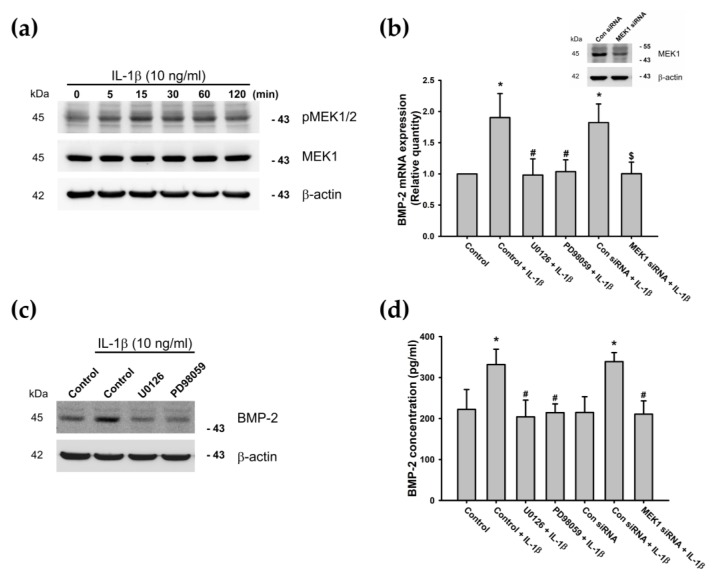
The MEK pathway is involved in IL-1β-induced BMP-2 expression. (**a**) ATDC5 cells were incubated with IL-1β (10 ng/mL) for the indicated times, and the phosphorylated MEK1/2 (pMEK1/2) was examined by Western blotting (n = 5). (**b**–**d**) ATDC5 cells were pretreated with MEK inhibitors (U0126 or PD98059) for 30 min or transfected with a MEK1 siRNA for 24 h, then stimulated with IL-1β for 24 h. BMP-2 expression was examined by qPCR (n = 5), Western blotting (n = 4), and ELISA (n = 6). Statistical testing was performed by one-way ANOVA. Quantification results are expressed as means ± SD. * *p* < 0.05 compared with the Control group; ^#^ *p* < 0.05 compared with the IL-1β-treated group; ^$^ *p* < 0.05 compared with the control siRNA-transfected group (Con siRNA) (**b**,**d**). Abbreviations: MEK: Mitogen-activated protein kinase; IL-1β: Interleukin 1 beta; BMP-2: Bone morphogenetic protein 2; pMEK1/2: Phosphorylated MEK1/2; siRNA: Small interfering RNA; qPCR: Real-time quantitative polymerase chain reaction; Con siRNA: Nontargeting Control siRNA.

Mitogen-activated protein kinases (MAPKs) are believed to play key regulatory roles in the production of various proinflammatory cytokines and downstream signaling events in processes leading to inflammation and articular cartilage destruction [71,72]. Therefore, we sought to determine whether MEK (a MAPK upstream activator) is involved in IL-1β-induced BMP-2 expression, by measuring levels of pMEK at different time intervals. The results revealed that IL-1β time-dependently enhanced MEK phosphorylation (Figure 3a). Pretreatment of ATDC5 cells with MEK inhibitors (U0126 and PD98059) or MEK siRNAs attenuated IL-1β-induced BMP-2 expression (Figure 3b–d).

**Figure 4:**
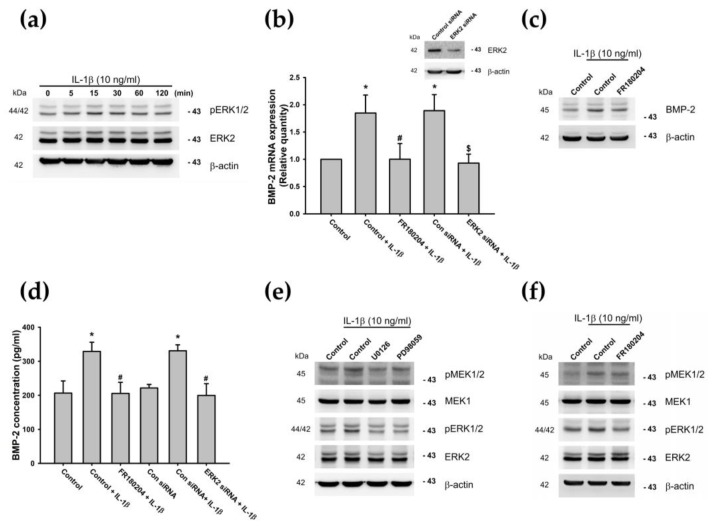
MEK-dependent ERK activation is involved in IL-1β-induced BMP-2 expression. (**a**) ATDC5 cells were incubated with IL-1β (10 ng/mL) for the indicated times, and phosphorylated ERK1/2 (pERK1/2) was examined by Western blotting (n = 5). (**b**–**d**) ATDC5 cells were pretreated with an ERK inhibitor (FR180204) for 30 min or transfected with an ERK2 siRNA for 24 h, then stimulated with IL-1β for 24 h. BMP-2 expression was examined by qPCR (n = 5), Western blotting (n = 4), and ELISA (n = 6). Statistical testing was performed by one-way ANOVA. (**e**) ATDC5 cells were pretreated with U0126 or PD98059 for 30 min and then stimulated with IL-1β for 15 min, and pMEK1/2, MEK1, pERK1/2, and ERK2 expression was examined by Western blotting (n = 4). (**f**) ATDC5 cells were pretreated with FR180204 for 30 min and then stimulated with IL-1β for 30 min, and pMEK1/2, MEK1, pERK1/2 and ERK2 expression was examined by Western blotting (n = 4). Quantification results are expressed as the mean ± SD. * *p* < 0.05 compared with the Control group; ^#^ *p* < 0.05 compared with the IL-1β-treated group; ^$^ *p* < 0.05 compared with the Con siRNA (**b**,**d**). Abbreviations: MEK: Mitogen-activated protein kinase; ERK: Extracellular signal-regulated kinase; IL-1β: Interleukin 1 beta; BMP-2: Bone morphogenetic protein 2; pERK1/2: Phosphorylated ERK1/2; siRNA: Small interfering RNA; qPCR: Real-time quantitative polymerase chain reaction; pMEK1/2: Phosphorylated MEK1/2; Con siRNA: Nontargeting Control siRNA.

Previous studies have suggested that IL-1β can be linked to the OA disease process by activating the MEK/ERK cascade [48,73,74]. Next, we examined ERK phosphorylation, to determine whether ERK signaling plays a role in IL-1β-induced BMP-2 expression. After 15 min of IL-1β (10 ng/mL) treatment, we found that levels of ERK phosphorylation increased in a time-dependent manner (Figure 4a). Conversely, IL-1β-induced BMP-2 expression was attenuated by pretreatment with an ERK inhibitor (FR180204) or by transfecting cells with ERK siRNAs (Figure 4b–d). Furthermore, we confirmed a relationship between the MEK and ERK signaling pathways. Incubating the ATDC5 cells with the MEK inhibitor reduced IL-1β-induced MEK and ERK phosphorylation (Figure 4e), but did not affect MEK phosphorylation when an ERK inhibitor was used (Figure 4f). Our data suggest that the MEK and ERK pathways are involved in IL-1β-induced BMP-2 expression in chondrocytes.

### 3.4. IL-1β-Induced Phosphorylation of Sp1 Through the MEK/ERK Signaling Pathway Promotes BMP-2 Expression

**Figure 5:**
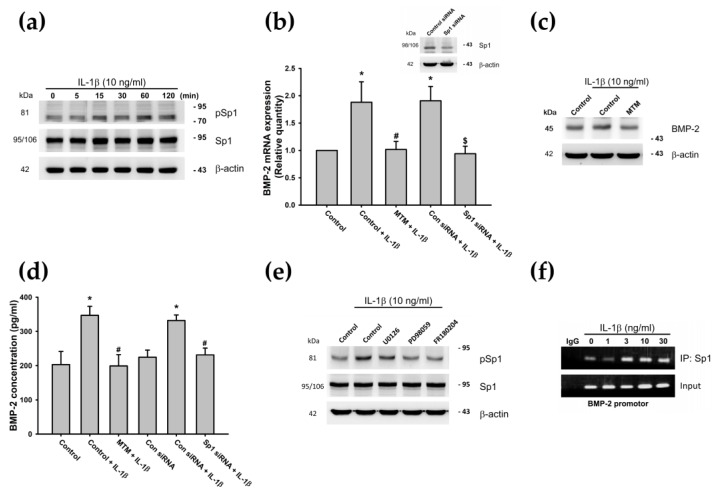
The MEK/ERK pathway is involved in IL-1β-induced Sp1 activation and BMP-2 expression. (**a**) ATDC5 cells were incubated with IL-1β (10 ng/mL) for the indicated times, and Sp1 phosphorylation was examined by Western blotting (n = 4). (**b**) ATDC5 cells were pretreated with the Sp1 inhibitor mithramycin A (MTM) for 2 h or transfected with a Sp1 siRNA for 24 h, then stimulated with IL-1β (10 ng/mL) for 24 h, and BMP-2 expression was evaluated by qPCR (n = 5). (**c**) ATDC5 cells were pretreated with MTM for 2 h, then stimulated with IL-1β (10 ng/mL) for 24 h. BMP-2 protein expression was examined by Western blotting (n = 5). Statistical testing was performed by one-way ANOVA. (**d**) ATDC5 cells were pretreated with MTM for 2 h or transfected with a Sp1 siRNA for 24 h, then stimulated with IL-1β (10 ng/mL) for 24 h, and BMP-2 expression was evaluated by ELISA (n = 5). Statistical testing was performed by one-way ANOVA. (**e**) ATDC5 cells were pretreated with U0126, PD98059, or FR180204 for 30 min and then stimulated with IL-1β for 60 min. pSp1 and Sp1 expression was examined by Western blotting (n = 4). (**f**) ATDC5 cells were incubated with different doses of IL-1β for 24 h and the ChIP assay was performed using the anti-Sp1 antibody (n = 3). Quantification results are expressed as the mean ± SD. * *p* < 0.05 compared with the Control group; ^#^ *p* < 0.05 compared with the IL-1β-treated group; ^$^ *p* < 0.05 compared with the control siRNA-transfected group stimulated with IL-1β (Con siRNA + IL-1β) (**b**,**d**). Abbreviations: MEK: Mitogen-activated protein kinase; ERK: Extracellular signal-regulated kinase; IL-1β: Interleukin 1 beta; Sp1: Specificity protein 1; BMP-2: Bone morphogenetic protein 2; siRNA: Small interfering RNA; qPCR: Real-time quantitative polymerase chain reaction; pSp1: Phosphorylated Sp1; Con siRNA: Nontargeting Control siRNA; IP: Immunoprecipitation.

The inhibition of Sp1 downregulates catabolic gene expression and reduces proinflammatory cytokine-induced production of ECM degradation enzymes [56,75]. Moreover, it appears that Sp1 stimulates the transcription of the BMP-2 gene [57]. Therefore, we examined whether Sp1 is involved in IL-1β-induced BMP-2 production, by analyzing Sp1 phosphorylation following IL-1β stimulation. The results demonstrated that treatment of ATDC5 cells with IL-1β (10 ng/mL) increased Sp1 phosphorylation in a time-dependent manner (Figure 5a). We also confirmed that pretreatment with the Sp1 inhibitor (mithramycin A) or transfection of cells with Sp1 siRNAs decreased IL-1β-induced BMP-2 mRNA levels and protein production (Figure 5b–d). To determine whether Sp1 is a downstream effector of the MEK/ERK pathway, the inhibitors of MEK (U0126 and PD98059) and ERK (FR180204) were used. Phosphorylation of Sp1 was observed in the Western blot analysis. Pretreatment with MEK or ERK inhibitors reduced IL-1β-induced Sp1 phosphorylation (Figure 5e). Moreover, we confirmed with the ChIP assay data that Sp1 binds to the BMP-2 promotor binding site after IL-1β (0, 1, 3, 10, or 30 ng/mL) stimulation, in a concentration-dependent manner (Figure 5f). These data suggest that IL-1β promotes BMP-2 expression in ATDC5 cells through the MEK/ERK/Sp1 signaling pathways.

### 3.5. Inhibition of BMP-2 Diminished Subchondral Bone Remodeling in Experimental OA

**Figure 6:**
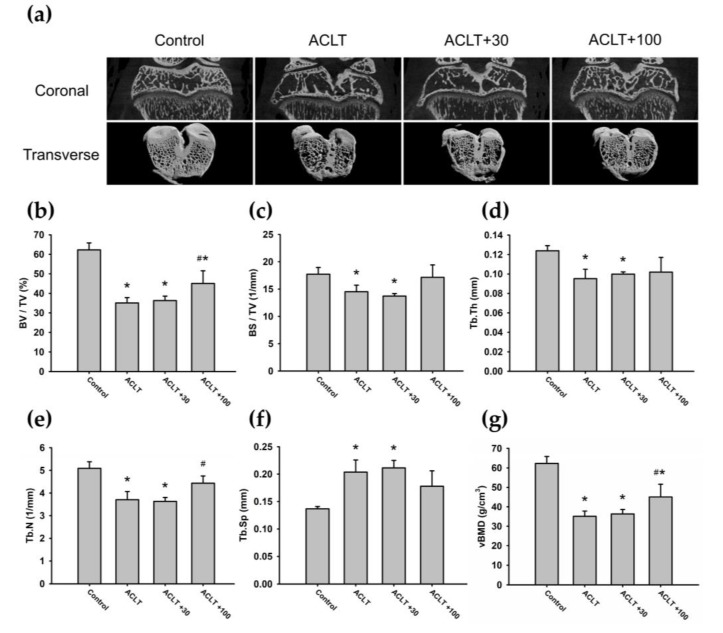
The BMP-2 inhibitor attenuates bone erosion and bone remolding in the experimental OA model. (**a**) Coronal and transverse (3D visualization) micro-CT images of proximal tibia in different groups (n = 6). (**b**) Bone volume fraction (BV/TV). (**c**) Bone surface density (BS/TV). (**d**) Trabecular thickness (Tb.Th). (**e**) Trabecular number (Tb.N). (**f**) Trabecular separation (Tb.Sp). (**g**) Volumetric bone mineral density (vBMD). All n = 5 in Figure 6b–g. All statistical testing was performed by one-way ANOVA, and quantification results are expressed as the mean ± SD. * *p* < 0.05 compared with the Control group; ^#^ *p* < 0.05 compared with the ACLT group. Abbreviations: BMP-2: Bone morphogenetic protein 2; OA: Osteoarthritis; micro-CT: Micro-computed tomography; ACLT: Anterior cruciate ligament transaction; BV/TV: Bone volume/tissue volume; BS/TV: Bone surface/tissue; ACLT+30: ACLT+30 ng Noggin protein; ACLT+100: ACLT+100 ng Noggin protein.

Then, we investigated whether BMP-2 inhibition could reverse the OA disease in vivo, using the rat ACLT model. Micro-CT analyses of coronal and transverse planes are shown in Figure 6a. All rats showed pathological tibial subchondral bone changes in the ACLT knee joint, particularly in the ACLT group and ACLT+30 group (Figure 6a). In each group, the ROI microarchitectural parameters were measured individually. The micro-CT data revealed significant reductions in the BV/TV fraction and vBMD in all ACLT animals compared with the control group (Figure 6b,g). Moreover, BS/TV, Tb.Th, and Tb.N measurements in the ACLT and ACLT+30 groups were significantly lower than those in the control group (Figure 6c–e). Furthermore, Tb.Sp was significantly increased in the ACLT group and ACLT+30 group compared with the control group (Figure 6f). Interestingly, there were no significant differences between the ACLT+100 group and control group for BS/TV, Tb.Th, Tb.N, and Tb.Sp measurements (Figure 6c–f). Furthermore, we found that the ACLT+100 group had higher BV/TV, Tb.N, and vBMD measurements compared with those in the ACLT and ACLT+30 groups (Figure 6b,e,g). Our findings suggest that OA-induced subchondral bone changes can be attenuated by intra-articular injections of BMP-2 inhibitors.

**Figure 7:**
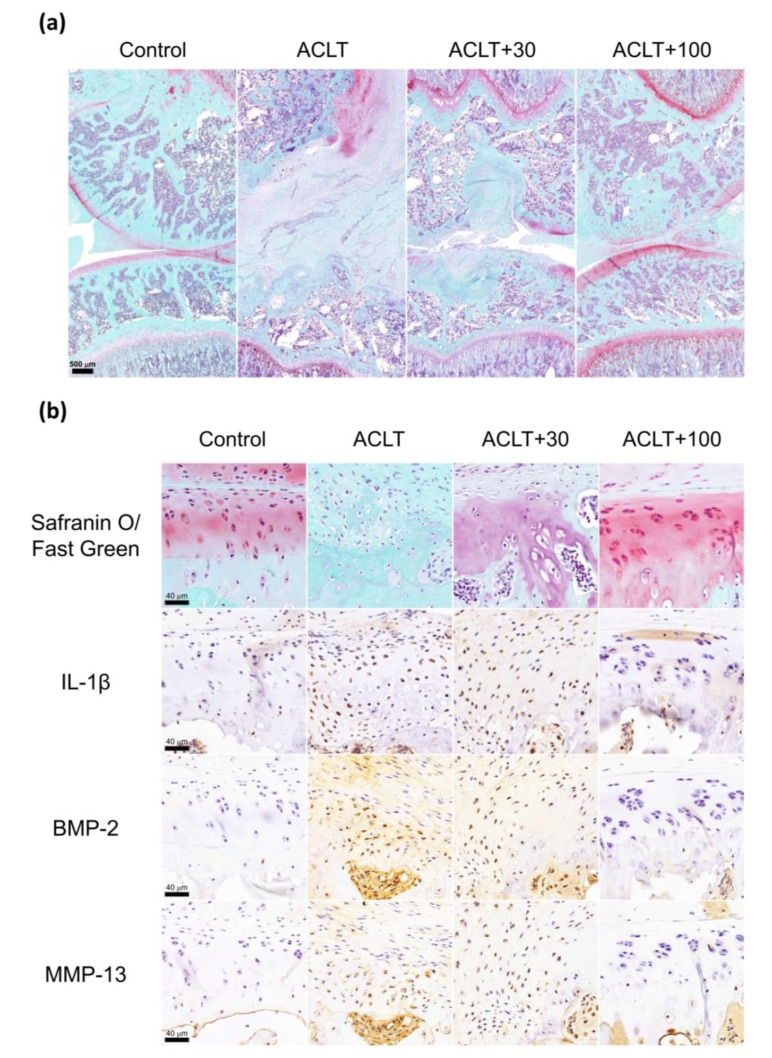
The experimental OA model increases cartilage degradation, as well as IL-1β, BMP-2, and MMP-13 expression in arthritic joints. (**a**) Paraffin sections of the knee joint from different groups were stained with Safranin O/Fast Green. Scale bar, 500 μm (n = 6). (**b**) Paraffin sections of the articular cartilage was IHC-stained with anti-IL-1β, anti-BMP-2, and anti-MMP-13. Scale bar, 40 μm (n = 6). Original magnification 63×. Images were obtained from digitized slides with the CaseViewer software. Abbreviations: IL-1β: Interleukin 1 beta; BMP-2: Bone morphogenetic protein 2; MMP-13: Matrix metalloproteinase 13; ACLT: Anterior cruciate ligament transaction; ACLT+30: ACLT+30 ng Noggin protein; ACLT+100: ACLT+100 ng Noggin protein.

Similarly, Safranin O/Fast Green staining revealed a significant articular cartilage erosion and destruction of the knee joint in the ACLT and ACLT+30 groups, as characterized by proteoglycan loss, chondrocyte clusters, and degenerative changes in the cartilage matrix (Figure 7a,b). Moreover, IHC staining of the knee joints revealed significantly increased levels of IL-1β, BMP-2, and MMP-13 expression in the ACLT and ACLT+30 groups; these levels were markedly diminished in the ACLT+100 group (Figure 7b).

## 4. Discussion

OA has long been considered as a degenerative disease of the cartilage, but increasing evidence indicates that chronic low-grade inflammation has a critical role in OA pathogenesis and is linked to OA progression [19,20]. Indeed, OA pathogenesis involves not only the degeneration and calcification of the cartilage, but also remodeling of the subchondral region. Many cytokines correlate with the severity of inflammation in OA joints and lead to an imbalance between catabolic and anabolic activity in joint tissues [22]. IL-1β is crucial for articular local inflammation in OA [19], and can independently induce inflammatory reactions and catabolism, degrade cartilage, promote chondrocyte hypertrophy, and inhibit ECM synthesis [25,39]. Although in vitro and in vivo investigations have established that IL-1β is a promising target for OA therapy, conflicting results have been observed from investigations into the inhibition of IL-1β- and IL-1β-deficient mice in OA models [21,76]. Therefore, it is necessary to consider whether the factors produced by chondrocytes in an inflammatory joint lead to the ossification of the cartilage and destruction of joint structure. Consistent with previous studies, our animal OA model shows high levels of IL-1β, BMP-2, and MMP-13 expression in the degenerated areas of the articular cartilage. Moreover, Safranin O/Fast Green staining shows degradation and calcification of cartilage in the ACLT group. In vitro studies have suggested that proinflammatory cytokines such as IL-1β and IL-6 are involved in calcification of the cartilage [25,77]. In addition, BMP-2 stimulates endochondral ossification of chondrocytes and interacts with MMP-13 to promote chondrocyte hypertrophy [32]. This indicates that a close relationship exists between inflammation and cartilage remodeling.

Recent investigations have highlighted the importance of homeostasis in tissue regeneration and destruction in OA [13]. Members of the BMP family perform important pleiotropic functions in regulating the development, homeostasis, and repair of various tissues. There is evidence that IL-6 induces BMP-2 vascular smooth muscle cell calcification in vitro, which mimics the process of bone formation [78]. Although proinflammatory cytokines such as IL-1β can induce BMP-2 expression in chondrocytes [39], few studies have explored the expression and function of BMPs in OA progression [32,79]. In patients with rheumatoid arthritis, stimulation with IL-1β induced BMP-2 and BMP-6, but not BMP-4, BMP-5, or BMP-7 in fibroblast-like synoviocytes [80,81]. In the OA cartilage, IL-1β was found to stimulate the expression of BMP-2, but not BMP-4 or BMP-6 in the cell and tissue explant culture [39]. In this study, we found that IL-1β stimulated BMP-2 expression in ATDC5 cells, which was detected in the conditioned medium.

IL-1β-induced activation of the MAPK pathway is involved in the expression of several proinflammatory factors and collagenase in chondrocytes [55,73,82]. The MEK/ERK signaling pathway is also related to chondrocyte hypertrophic differentiation and fibrocartilage formation [82]. The use of MEK inhibitors alone or in combination with ERK inhibitors reduces chondrocyte hypertrophy and matrix resorption, and slows the process of cartilage remodeling [74,83]. Research has also indicated that inhibiting the MEK-ERK1/2 pathway decreases the development of the structural changes in a rabbit model of OA [84]. In our present study, inhibitors of MEK (U0126 and PD98059), ERK (FR180204), and siRNAs against MEK or ERK all blocked the IL-1β-induced BMP-2 production. These results suggest that MEK and ERK activation is required for BMP-2 production in chondrocytes.

Sp1 plays an important role in the regulation of apoptosis and fibrosis, and can directly promote the transcription of BMP-2 [57]. Moreover, previous research has suggested that Sp1 is important to regulating gene expression in the cartilage, and for mediating the biological responses of chondrocytes to inflammatory cytokine stimulation [56,75,85]. Sp1 overexpression enhances the IL-1-induced aggrecanase expression and activity [56]. Inhibiting Sp1 downregulates proinflammatory cytokine-induced MMP expression in the articular chondrocytes [75]. In another study, osteogenic profile markers were downregulated in vivo and in vitro by inhibition or gene silencing of Sp1 [57], while other research has suggested that Sp1 inhibition blocks the resorption of bone and cartilage via multiple mechanisms [75]. In this present study, inhibition of Sp1 DNA binding by a Sp1 inhibitor (mithramycin A) or gene silencing by Sp1 siRNAs significantly decreased the IL-1β-induced BMP-2 production in ATDC5 cells. Many inducers can induce the phosphorylation of Sp1, such as growth factors [86] and proinflammatory cytokines [87]. The phosphorylated state of Sp1 influences its transcriptional activity in different cell models [86,88]. The epidermal growth factor and fibroblast growth factor-2 have been found to induce phosphorylation of Sp1 via the activation of the MEK/ERK signaling pathway [86]. IL-1β increases Sp1 phosphorylation and activity in synovial fibroblasts [87]. In the present study, the Western blot analysis revealed that IL-1β promotes Sp1 phosphorylation through the MEK/ERK pathway. Furthermore, the ChIP assay showed that IL-1β induces BMP-2 expression through the binding of Sp1 to the BMP-2 promoter region. However, the detailed role of Sp1 in OA pathogenesis needs further examination.

An appropriate balance between anabolic and catabolic activities in articular chondrocytes contributes to maintenance of the existing ECM in the healthy cartilage [32,89]. However, this balance is lost in OA, where tissue degradation outweighs the capacity of tissue repair and leads to cartilage degradation [31,32]. In early OA, elevated BMP-2 levels in the damaged cartilage can both induce tissue repair by increasing ECM synthesis and cartilage degradation by stimulating MMP-13 expression [26,35]. Unfortunately, this response fails to repair the cartilage because of the higher level of catabolism [32]. Increased inflammation and production of connective tissue degrading enzymes contribute to further cartilage degradation in OA. Chondrocytes change to a degradative and hypertrophic-like state with OA progression. This hypertrophic differentiation is similar to endochondral ossification in the growth plate during development [26,89]. In addition, chondro-osteogenesis is a complex process that is regulated by an interaction between BMPs and Noggin protein [31,32]. Articular chondrocytes do not undergo hypertrophy in healthy cartilage [31,32]. The strong BMP stimulation of chondrocyte terminal differentiation (hypertrophy), involving abnormal ECM production (collagen type X), increased activity of MMP-13, and promotion of BMP-related cartilage-subchondral plate remodeling, initiates the OA pathogenesis [26,32,89].

Although the BMP-2 treatment in skeletal defects has demonstrated a high potential for bone formation, abnormal bone remodeling and inflammation has been found after high doses of BMP-2 in clinical applications [32,90]. Our micro-CT imaging revealed significant focal fragmentation and intersecting bone trabeculae areas in the experimental OA model. The significant decrease in Tb.Th, Tb.N, and vBMD, and increase in Tb.Sp, revealed structural changes in the subchondral bone. Previous studies have found that early-stage OA is associated with bone loss via increased bone remodeling [91]. Moreover, increased turnover of subchondral bone is a determinant of OA progression, and bone resorption is increased in progressive OA compared with nonprogressive OA [92].

Noggin blocks the BMP-2 function by preventing BMP-2 from binding with its receptors [42,43]. However, there is little research on the intra-articular injection of exogenous Noggin protein as an inhibitor of BMP-2. Previous studies have indicated that the optimal concentration of intrathecal Noggin injection in rats is 2 μg/mL, but it seems to also work at 10 ng/mL [63]. Another study showed that the intra-articular injection of gremlin-1 (a BMP inhibitor) at 1 μg/mL(lowest dosage) twice a week for eight weeks can also reduce BMP signaling in the cartilage [64]. Therefore, we chose two different concentrations (30 ng (0.6 μg/mL) and 100 ng (2 μg/mL)) to test the inhibitory effect of exogenous Noggin on BMP-2. Our study found that the inhibition of BMP-2 in the experimental OA model decreases subchondral bone destruction in the tibial plateau. The intra-articular injection of Noggin reduced the expression of IL-1β, BMP-2, and MMP-13. This suggests that lowering BMP-2 expression reduces structural remodeling and inflammation in the articular cartilage. Other research has reported that the inhibition of TGF-β activity in the subchondral bone attenuates articular cartilage degeneration and suppresses subchondral bone remodeling in the ACLT mouse model of OA [93]. This implies that regulating the cartilage-subchondral bone remodeling process includes other growth factors. However, we did not examine the expression of all BMP family members in the cartilage after intra-articular injection of Noggin.

Inflammation is intimately linked to the remodeling of the cartilage and the subchondral area, which plays a central role in OA progression. Therefore, antagonism of BMP signaling is an attractive therapeutic principle. In the present study, we examined the role of IL-1β in BMP-2 expression using the chondrocytic ATDC5 cell line. Our results suggest that IL-1β promotes BMP-2 expression in chondrocytes through the MEK, ERK, and Sp1 signaling pathways. Inhibition of BMP-2 activity by intra-articular injection of Noggin decreased cartilage degradation and also the extent of subchondral structure remodeling. Inflammatory mediators released by the articular cartilage may lead to subchondral bone loss by increasing structural changes in OA. The findings in this study provide new insights into the possible mechanism of IL-1β-induced BMP-2 expression in chondrocytes and OA development. Targeting pathologic bone remodeling and inflammation with the use of Noggin or similar drugs could represent a potential therapeutic approach to treat OA.
